# Autoantibodies Neutralizing Type I IFNs in the Bronchoalveolar Lavage of at Least 10% of Patients During Life-Threatening COVID-19 Pneumonia

**DOI:** 10.1007/s10875-023-01512-9

**Published:** 2023-05-20

**Authors:** Quentin Philippot, Arnaud Fekkar, Adrian Gervais, Tom Le Voyer, Leonoor S. Boers, Clément Conil, Lucy Bizien, Justin de Brabander, Jan Willem Duitman, Alessia Romano, Jérémie Rosain, Marion Blaize, Mélanie Migaud, Maxime Jeljeli, Boualem Hammadi, Aurore Desmons, Astrid Marchal, ArtDECO consortium, Esther J. Nossent, Anno Saris, Heder De Vries, Lilian J. Meijboom, Siebe G. Blok, Alex R. Schuurman, Tom D. Y. Reijnders, F. Hugenholtz, Juan J. Garcia Vallejo, Hetty Bontkes, Alexander P. J. Vlaar, Joost Wiersinga, René Lutter, Tom van der Poll, Harm Jan Bogaard, Robert F. J. Kullberg, Shiqi Zhang, Esther J. Nossent, Leo M. A. Heunks, Pieter Roel Tuinman, Peter I. Bonta, COVID HGE consortium, Laurent Abel, Saleh Al-Muhsen, Andrés A. Arias, Dusan Bogunovic, Alexandre Bolze, Ahmed A. Bousfiha, Davood Mansouri, Isabelle Meyts, Rebeca Perez de Diego, Vanessa Sancho-Shimizu, András N. Spaan, Stuart G. Tangye, Shen-Ying Zhang, Helen C. Su, Julien Mayaux, Qian Zhang, Emmanuelle Jouanguy, Raphael Borie, Bruno Crestani, Charles Edouard Luyt, Homa Adle-Biassette, Damien Sene, Bruno Megarbane, Aurélie Cobat, Paul Bastard, Lieuwe D. J. Bos, Jean-Laurent Casanova, Anne Puel

**Affiliations:** 1 Laboratory of Human Genetics of Infectious Diseases, Imagine Institute for Genetic Diseases, Necker Branch, INSERM U1163, Necker Hospital for Sick Children, 24 Boulevard du Montparnasse 75015, EU Paris, France; 2 Imagine Institute, Université Paris Cité, Paris, EU France; 3 AP-HP, Groupe Hospitalier La Pitié-Salpêtrière, Service de Parasitologie Mycologie, Paris, EU France; 4 Amsterdam UMC, University of Amsterdam, Intensive Care Medicine, Meibergdreef 9, Amsterdam, EU The Netherlands; 5 Center for Experimental Molecular Medicine, Amsterdam UMC, Amsterdam, EU Netherlands; 6 Amsterdam UMC, Location AMC, Department of Pulmonary Medicine, University of Amsterdam, 1105 AZ Amsterdam, EU The Netherlands; 7 Amsterdam UMC, Department of Experimental Immunology, Location University of Amsterdam, 1105 AZ Amsterdam, EU The Netherlands; 8 Amsterdam Infection & Immunity, Inflammatory Diseases, 1105 AZ Amsterdam, EU The Netherlands; 9 Département 3I « Infection, Immunité Et Inflammation », Institut Cochin, INSERM U1016, Université Paris Cité, Paris, EU France; 10 Faculté de Médecine, AP-HP-Centre Université de Paris, Hôpital Cochin, Service d’Immunologie Biologique, Université Paris Cité, Paris, EU France; 11 General Chemistry Laboratory, Department of Clinical Chemistry, APHP, Necker Hospital for Sick Children, Paris, EU France; 12 Clinical Metabolomic Department, Sorbonne Université, INSERM, Centre de Recherche Saint-Antoine (CRSA), Saint Antoine Hospital, Assistance Publique des Hôpitaux de Paris (AP-HP Sorbonne Université), Paris, France; 13 INSERM, UMRS1158 Neurophysiologie Respiratoire Expérimentale et Clinique, Sorbonne Université, Paris, EU France; 14 Site Pitié-Salpêtrière, Service de Pneumologie, Médecine Intensive et Réanimation, Département R3S, Hôpital Pitié-Salpêtrière, AP-HP, Sorbonne Université, Paris, EU France; 15 St. Giles Laboratory of Human Genetics of Infectious Diseases, Rockefeller Branch, Rockefeller University, New York, NY USA; 16 Service de Pneumologie A Hôpital Bichat, APHP, Paris, EU France; 17 Inserm, PHERE, Université Paris Cité, 75018 Paris, EU France; 18 Service de Médecine Intensive Réanimation, Institut de Cardiologie, AP-HP, Hôpital Pitié-Salpêtrière, Paris, EU France; 19 Inserm, Institute of Cardiometabolism and Nutrition (ICAN), Sorbonne Université, Paris, EU France; 20 AP-HP, Hôpital Lariboisière, Service Anatomie Pathologique and Université de Paris, Paris, EU France; 21 Inserm, NeuroDiderot, Paris, EU France; 22 Internal Medicine Department, AP-HP, Lariboisière Hospital, Paris, EU France; 23 Université Paris Cité, Paris, EU France; 24 Department of Medical and Toxicological Critical Care, APHP, Lariboisière Hospital, Paris, EU France; 25 INSERM UMRS-1144, Paris-University, Paris, EU France; 26 Pediatric Hematology-Immunology and Rheumatology Unit, Necker Hospital for Sick Children, AP-HP, Paris, France; 27 Department of Pediatrics, Necker Hospital for Sick Children, Paris, EU France; 28 Howard Hughes Medical Institute, New York, NY USA

**Keywords:** COVID-19, SARS-CoV-2, Cytokines, Type I interferons

## Abstract

Autoantibodies (auto-Abs) neutralizing type I interferons (IFNs) are found in the blood of at least 15% of unvaccinated patients with life-threatening COVID-19 pneumonia. We report here the presence of auto-Abs neutralizing type I IFNs in the bronchoalveolar lavage (BAL) of 54 of the 415 unvaccinated patients (13%) with life-threatening COVID-19 pneumonia tested. The 54 individuals with neutralizing auto-Abs in the BAL included 45 (11%) with auto-Abs against IFN-α2, 37 (9%) with auto-Abs against IFN-ω, 54 (13%) with auto-Abs against IFN-α2 and/or ω, and five (1%) with auto-Abs against IFN-β, including three (0.7%) with auto-Abs neutralizing IFN-α2, IFN-ω, and IFN-β, and two (0.5%) with auto-Abs neutralizing IFN-α2 and IFN-β. Auto-Abs against IFN-α2 also neutralize the other 12 subtypes of IFN-α. Paired plasma samples were available for 95 patients. All seven patients with paired samples who had detectable auto-Abs in BAL also had detectable auto-Abs in plasma, and one patient had auto-Abs detectable only in blood. Auto-Abs neutralizing type I IFNs are, therefore, present in the alveolar space of at least 10% of patients with life-threatening COVID-19 pneumonia. These findings suggest that these auto-Abs impair type I IFN immunity in the lower respiratory tract, thereby contributing to hypoxemic COVID-19 pneumonia.

**Supplementary Information:**

The online version contains supplementary material available at 10.1007/s10875-023-01512-9.

## Introduction

SARS-CoV-2 infection displays immense interindividual clinical variability in unvaccinated individuals, ranging from silent infection to lethal disease [1, 2]. Silent or mild infection is seen in about 80% of individuals, while pneumonia strikes about 20% of cases, with half these cases displaying hypoxemic pneumonia and one third acute respiratory distress syndrome (ARDS) [1–3]. Global mortality is about 0.5–1%, across all ages and sexes, with a risk of death that doubles every five years of age, from childhood onward [4]. Sex, comorbid conditions, and common human genetic variants have been identified as risk factors, but have a lesser effect than age, with odds ratios typically < 1.5 and always < 2 [1, 2]. Inborn errors of type I interferon (IFN) immunity or autoantibodies (auto-Abs) against type I IFNs account for at least 15–20% of cases of life-threatening COVID-19 pneumonia [5–10]. Circulating auto-Abs against type I IFNs can neutralize high (10 ng/mL) or low and more physiological (100 pg/mL) concentrations of type I IFNs [8]. These auto-Abs have been found in at least 15% of patients with critical COVID-19 pneumonia [7, 8]. They have also been found in ~ 20% of cases of life-threatening COVID-19 pneumonia in patients over the age of 80 years, and in ~ 20% of patients with fatal COVID-19 across all ages [8]. They have been shown to neutralize the 12 different IFN-α subtypes and/or IFN-ω, and, more rarely, IFN-β [8]. These auto-Abs are associated with life-threatening pneumonia, with ORs increasing with the number and concentration of type I IFNs neutralized (OR values ranging from 3 to 67) [8, 11]. These findings have been confirmed in 29 other studies [12–40]. These auto-Abs have also been detected in the plasma/serum of individuals from the general population collected before SARS-CoV-2 infection, in ~ 0.2% (neutralizing high concentration of type I IFNs) and 1% (neutralizing low concentration of type I IFNs) of individuals aged 18 to 69 years, 1% and 2.3%, respectively, of those aged between 70 and 80 years, and their frequency reached 3.4% and 6.3%, respectively, in individuals over the age of 80 years [8]. Life-threatening COVID-19 pneumonia in patients with auto-Abs against type I IFNs may, therefore, be considered an autoimmune condition, with adaptive B-cell immunity disrupting innate type I IFN-dependent immunity [41].

Patients with life-threatening COVID-19 pneumonia, with or without auto-Abs against type I IFNs, display pulmonary and systemic inflammation [2]. This suggested a two-step model, in which insufficient type I IFN production or responses to type I IFNs in the first few days of infection allow the virus to spread from the upper to the lower respiratory tract, and to various tissues via the bloodstream. This viral dissemination triggers the recruitment and activation of leukocytes, unleashing excessive inflammation from the second week of infection onward [1, 2]. The nasopharyngeal mucosa is the port of entry of SARS-CoV-2. In patients with mild, upper respiratory tract COVID-19 without pneumonia, the levels of type I and type III (I/III) IFN-dependent interferon-stimulated gene (ISG) induction in this mucosa are correlated with serum IFN-α2 concentration and nasal SARS-CoV-2 load [42]. In patients with critical COVID-19 pneumonia, the induction of type I/III IFN-dependent ISGs in the nasopharyngeal mucosa is weaker in patients with auto-Abs against type I IFNs than in those without such antibodies [42]. Following SARS-CoV-2 infection, pre-existing auto-Abs against type I IFNs in the blood probably contribute to viral spread, via the bloodstream, to various tissues [1, 2]. By contrast, the contribution of auto-Abs against type I IFNs to the spread of the virus from the upper to the lower respiratory tract remains unclear. Single-cell transcriptomic studies of the bronchoalveolar lavage (BAL) of patients with critical COVID-19 pneumonia found impaired type-I IFN signaling in the T cells and alveolar macrophages relative to patients with moderate or severe COVID-19 pneumonia [43]. Type I IFN-dependent immunity may, therefore, contribute to alveolar defenses against SARS-CoV-2. Auto-Abs against type I IFNs have been detected in tracheal aspirate [44]. However, their presence and neutralizing activity in the alveolar space have been assessed in only 11 individuals and demonstrated in only three of these individuals [21]. In this study, we aimed to plug this gap in current knowledge by testing for the presence of neutralizing auto-Abs against type I IFNs in the BAL fluid of a large cohort of patients with life-threatening COVID-19 pneumonia.

## Methods

### Study Design

We enrolled 415 patients with proven life-threatening COVID-19 from three university hospitals in France and the Netherlands. We collected BAL from all these patients, and plasma from a subset of 95 patients (collected within 24 h of the BAL sample), for immunoassays to assess the presence of IgG auto-Abs against type I IFNs. All individuals were recruited according to protocols approved by local institutional review boards (IRBs).

### Detection of Anti-Cytokine Auto-Abs

Gyros was used for the detection of anti-type I IFN auto-Abs, as described by Bastard et al*.* [8]. Cytokines, recombinant human (rh)IFN-α2 (Miltenyi Biotec, reference number 130–108-984) and rhIFN-ω (Merck, reference number SRP3061) were first biotinylated with EZ-Link Sulfo-NHS-LC-Biotin (Thermo Fisher Scientific, catalog number A39257), according to the manufacturer’s instructions, with a biotin-to-protein molar ratio of 1:12. The detection reagent contained a secondary antibody [Alexa Fluor 647 goat anti-human IgG (Thermo Fisher Scientific, reference number A21445)] diluted in Rexxip F (Gyros Protein Technologies, reference number P0004825; 1:500 dilution of the 2 mg/mL stock to yield a final concentration of 4 μg/mL). Phosphate-buffered saline, 0.01% Tween 20 (PBS-T) and Gyros Wash buffer (Gyros Protein Technologies, reference number P0020087) were prepared according to the manufacturer’s instructions. BAL or plasma samples were then diluted 1:100 in 0.01% PBS-T and tested with the Bioaffy 1000 CD (Gyros Protein Technologies, reference number P0004253) and the Gyrolab xPand (Gyros Protein Technologies, reference number P0020520). Cleaning cycles were performed in 20% ethanol.

### Functional Evaluation of Anti-Type I IFN Auto-Abs in Luciferase Reporter Assays

The neutralizing activity of anti–IFN-α2 and anti–IFN-ω auto-Abs was assessed in a reporter luciferase activity, as described by Bastard et al*.* [8]. HEK293T cells were transfected with a plasmid containing the firefly luciferase gene under the control of the human ISRE promoter in the pGL4.45 backbone and a plasmid constitutively expressing the *Renilla* luciferase for normalization (pRL-SV40). The cells were transfected in the presence of the X-tremeGENE9 transfection reagent (Sigma-Aldrich, reference number 6365779001) for 24 h. Cells in Dulbecco’s modified Eagle medium (DMEM; Thermo Fisher Scientific) supplemented with 2% fetal calf serum and 10% patient plasma or 20% BAL (after inactivation at 56 °C for 20 min) were stimulated with IFN-α2 (Miltenyi Biotec, reference number 130–108-984) or IFN-ω (Merck, reference number SRP3061), at 10 ng/mL or 100 pg/mL, or rhIFN-β (Peprotech, ref. number 300-02BC) at 10 ng/mL for 16 h at 37 °C. Each sample was tested once for each cytokine and each dose. Finally, the cells were lysed for 20 min at room temperature, and luciferase levels were measured with the Dual-Luciferase Reporter 1000 Assay System (Promega, reference number E1980) according to the manufacturer’s protocol. Luminescence intensity was measured with a VICTOR-X Multilabel Plate Reader (Perkin Elmer Life Sciences, USA). Firefly luciferase activity was normalized against *Renilla* luciferase activity. A similar protocol was used to test for auto-Abs against 12 subtypes of IFN-α, except that we used cytokines from PBL Assay Science (catalog no. 11002–1) at a concentration of 1 ng/mL for stimulation.

### IgG Purification

We demonstrated that the IFN-α2– or IFN-ω–neutralizing activity observed was due to auto-Abs and not another BAL factor, by depleting IgG from the BAL with a protein G buffer (Pierce Protein G IgG Binding Buffer, 21,011) and column (NAb Protein G Spin Columns, 89,953). All buffers were prepared in the laboratory: 0.1 M glycine (pH 2.7) and 1.5 M Tris (pH 8). Total BAL was loaded onto the column. Each sample was tested once. Purified IgG was then concentrated [Pierce Protein Concentrators polyethersulfone (PES), 50 K molecular weight cut-off (MWCO), 88504]. The flow-through fraction (IgG-depleted) was collected without eluting IgG and compared with total BAL in the luciferase neutralization assay.

### Assessment of Urea and Hemoglobin Concentrations in the BAL

Urea and hemoglobin concentrations were assessed in the BAL with the Urea Assay Kit and the Hemoglobin Assay Kit, both from Sigma (MAK006 and MAK115, respectively), according to the manufacturer’s guidelines.

### Determination of Biomarker Concentrations in the BAL

Cytokine and chemokine concentrations were measured with a Luminex multiplex assay (R&D Systems) on a BioPlex200 (BioRad), as previously described [45].

### Statistical Analysis

Analyses were performed in R v4.0.5 GUI 1.74 or in GraphPad Prism 8.4.3.

## Results

### Auto-Abs Neutralizing IFN-α2 and/or IFN-ω in the Plasma of 17% of Patients with Life-Threatening COVID-19 Pneumonia

**Fig. 1:**
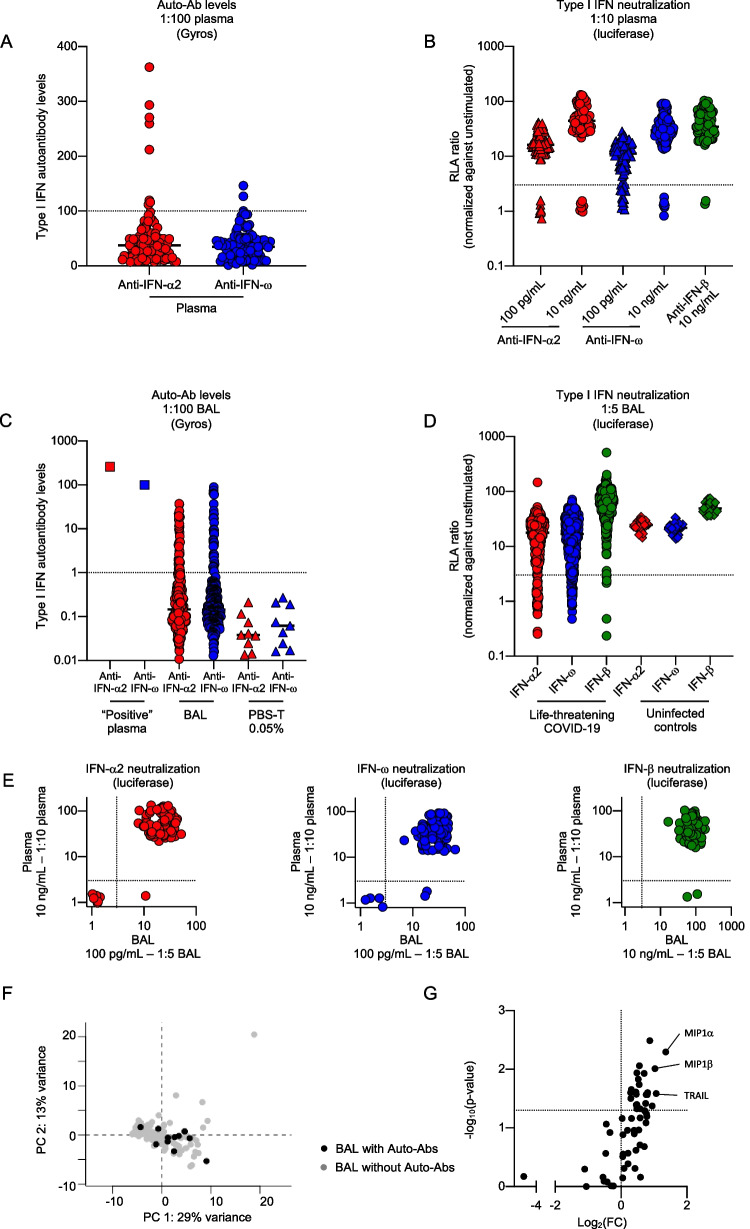
Autoantibodies neutralizing type I interferons are present in the broncho-alveolar lavage of ~ 10% of patients with life-threatening COVID-19. (A) Gyros (high-throughput automated ELISA) results for auto-Abs against IFN-α2 and/or IFN-ω in the plasma of patients with life-threatening COVID-19 (*N* = 95). The dotted line indicates the threshold for positivity, defined as a type I IFN auto-Ab level exceeding 100. (B) Results for the neutralization of IFN-α2, IFN-ω (100 pg/mL or 10 ng/mL) or IFN-β (10 ng/mL) in the presence of a 1:10 dilution of plasma from patients with life-threatening COVID-19 (*N* = 95). The relative luciferase activity (RLA) ratio (ISRE dual luciferase activity, with normalization against *Renilla* luciferase activity) is shown, after stimulation with IFN-α2, IFN-ω, or IFN-β, with normalization against the RLA obtained without stimulation in the presence of a 1:10 dilution of plasma. The dotted line indicates the threshold for neutralization, defined as an RLA ratio of no more than 3. (C) Gyros results for auto-Abs against IFN-α2 and IFN-ω in the plasma of a patient known to have high titers of auto-Abs neutralizing IFN-α2 and IFN-ω (“Positive” plasma), in the bronchoalveolar lavage (BAL) of patients with life-threatening COVID-19 (*N* = 254), and in PBS supplemented with 0.05% Tween (PBS-T) as a negative control. The dotted line indicates the threshold for positivity, defined as a type I IFN auto-Ab level above 1. (D) Results for the neutralization of IFN-α2, IFN-ω (100 pg/mL), or IFN-β (10 ng/mL) in the presence of a 1:5 dilution of BAL from patients with life-threatening COVID-19 (*N* = 415) and individuals without interstitial lung disease (“Uninfected controls” – *N* = 27). Relative luciferase activity (RLA) ratio (ISRE dual luciferase activity, with normalization against *Renilla* luciferase activity) is shown after stimulation with IFN-α2, IFN-ω, or IFN-β, with normalization against the RLA without stimulation in the presence of a 1:5 dilution of BAL. The dotted line indicates the threshold for neutralization, defined as an RLA ratio of no more than 3. (E) Plot showing the RLA ratio after stimulation with IFN-α2 or IFN-ω or IFN-β, in the presence of a 1:10 dilution of plasma or a 1:5 dilution of BAL from patients with life-threatening COVID-19 (*N* = 95). The dotted lines indicate the threshold for neutralization, defined as an RLA ratio of no more than 3. Patients with neutralizing auto-Abs in both BAL and plasma are shown in the bottom left corner, whereas the patients in the bottom right corner had neutralizing auto-Abs only in plasma. (F-G) Principal component analysis (PCA) (F), and volcano plot representation (G) of the concentrations of 59 biomarkers in BAL from patients with life-threatening COVID-19 pneumonia with (*N* = 11) or without (*N* = 117) auto-Abs against type I IFNs. PCA was performed with the FactoMineR package. Wilcoxon tests were performed to compare the concentrations of biomarkers in BAL between patients with and without auto-Abs against type I IFNs, with a *p*-value < 0.05 considered significant

We recruited an international cohort of 415 patients with life-threatening COVID-19 pneumonia from three university hospitals: the *La Pitié-Salpêtrière* (*N* = 259, 62%) and *Lariboisière* (N = 32, 8%) hospitals, both part of the *Assistance Publique-Hôpitaux de Paris* (*AP-HP*) network in Paris, France, and Amsterdam University Medical Centers (UMC) (*N* = 124, 30%) in Amsterdam, the Netherlands. All these patients were hospitalized in an intensive care unit with invasive ventilation. They had a median age of 60 years [50 – 67 years] and 69% were men. Overall mortality was 51% (Table 1 and Figure S1A). We tested for auto-Abs against IFN-α2 and/or IFN-ω in plasma samples, which were available for 95 (mean age 65 years, 72% men) individuals from this cohort. We used Gyros Technology, a high-throughput automated enzyme-linked immunosorbent assay (ELISA)-like assay that we have validated for the detection of circulating anti-IFN-α2 or anti-IFN-ω immunoglobulin G (IgG) [8]. Eight (8%) and three (3%) patients had high levels (> 100) of anti-IFN-α2 and anti-IFN-ω IgG, respectively, and one (1%) patient had high levels of IgG against IFN-α2 and IFN-ω (Fig. 1A). We assessed the ability of these auto-Abs to neutralize high (10 ng/mL), or low (100 pg/mL), more physiological concentrations of type I IFNs in a 1:10 dilution of plasma. We used a previously described neutralization assay developed in HEK293T cells transfected with a luciferase system [8]. Eight of the 95 individuals tested (8%) had auto-Abs neutralizing high concentrations of IFN-α2 and/or IFN-ω, 16 (17%) had auto-Abs neutralizing low concentrations of IFN-α2 and/or IFN-ω, and two (2%) had auto-Abs neutralizing high concentrations of IFN-α2, IFN-ω, and IFN-β (Fig. 1B and Table 2).

**Table 1:** Clinical characteristics of the patients included in this cohort

	All patients - *N*=415	Patients without auto-Abs in BAL - *N*=361	Patients with auto-Abs in BAL - *N*=54	*P*-value^*^	Missing data
Age (years)	60 (50—67)	59 (50—67)	63 (57 – 71)	0.0045	10 (2%)
Male	277 (69%)	241 (68%)	36 (73%)	0.46	13 (3%)
Death	202 (51%)	172 (50%)	30 (60%)	0.19	19 (5%)
Length of ICU stay (days)	28 (16 – 47)	29 (16 – 47)	24 (12 – 48)	0.69	209 (50%)
Duration of invasive ventilation (days)	25 (13 – 42)	26 (14 – 42)	24 (12 – 45)	0.79	216 (52%)
Time from symptom onset to BAL (days)	15 (9 – 21)	15 (9 – 21)	14 (10 – 24)	0.79	330 (80%)
Time from ICU admission to BAL (days)	7 (4—12)	7 (4—13)	6 (4—10)	0.22	214 (52%)

Data are reported as *N*, *N* (%), or median (interquartile range); *Patients with autoantibodies against type I IFNs (auto-Abs) in bronchoalveolar lavage (BAL) were compared with those without such antibodies, in *t*- or chi-squared tests, with a *P*-value < 0.05 considered significant

**Table 2:** Number and proportion of patients with auto-Abs neutralizing type I IFNs in the plasma

Type and concentration of type I IFNs neutralized by plasma diluted 1:10	Number (%) of patients with neutralizing activity
Anti–IFN-α2 and/or anti–IFN-ω auto-Abs (10 ng/mL)	8 (8%)
Anti–IFN-α2 and anti–IFN-ω auto-Abs (10 ng/mL)	6 (6%)
Anti–IFN-α2 auto-Abs (10 ng/mL)	8 (8%)
Anti–IFN-ω auto-Abs (10 ng/mL)	6 (6%)
Anti–IFN-α2 and/or anti–IFN-ω auto-Abs (100 pg/mL)	16 (17%)
Anti–IFN-α2 and anti–IFN-ω auto-Abs (100 pg/mL)	7 (7%)
Anti–IFN-α2 auto-Abs (100 pg/mL)	8 (8%)
Anti–IFN-ω auto-Abs (100 pg/mL)	15 (16%)
Anti–IFN-β auto-Abs (10 ng/mL)	2 (2%)
Anti–IFN-α-2, anti–IFN-ω and anti–IFN-β auto-Abs (10 ng/mL)	2 (2%)

### Detection of Anti-IFN-α2 and Anti-IFN-ω IgG Auto-Abs in the BAL of Patients With Life-Threatening COVID-19 Pneumonia

We then searched for auto-Abs against type I IFNs in BAL. As IgG, but not IgA, anti-GM-CSF auto-Abs had already been described in the BAL [46], we used Gyros technology to search for IgG auto-Abs against IFN-α and IFN-ω in BAL samples from the patients. Taking into account the dilution of the lung alveolar epithelial lining fluid (ELF) in the BAL (previously reported to be ~ 100-fold [47] and estimated at 94- to 302-fold in five BAL samples from our cohort – Table S1), we considered auto-Ab levels above background (defined as level > 1) to be “positive”. Using this threshold, we tested the BAL samples from 254 of the 415 patients of the cohort (mean age 59 years, 71% men) and found that 41 (16%) and 37 (15%) patients had anti-IFN-α2 and anti-IFN-ω IgG, respectively, in their BAL (Fig. 1C). IgG auto-Abs against both IFN-α2 and IFN-ω were found in the BAL of 25 patients (10%). The hemoglobin concentrations of the BAL samples tested did not differ between BAL with and without anti-type I IFN IgG, and were below those in BAL from patients with cytologically diagnosed alveolar hemorrhage (Figure S1B), suggesting that these auto-Abs were present in the alveolar space in the absence of alveolar hemorrhage or bronchial hemorrhage related to bronchoscopy. We assessed the neutralizing capacity of these auto-Abs in BAL, using HEK293T cells in a luciferase neutralization assay in which the cells were incubated with medium containing a “negative” BAL (i.e. no anti-IFN-α2 and no anti-IFN-ω IgG detected in the BAL or in the corresponding plasma by Gyros) diluted 1:5. This “negative” BAL did not significantly impair luciferase induction. The neutralizing activity of an anti-human IFN-α2 monoclonal IgG was not impaired when incubated with this BAL (Figure S1C). We then used this system to assess the neutralizing activity of one “positive” BAL (i.e. anti-IFN-α2 and anti-IFN-ω IgG detected in the BAL and in the corresponding plasma by Gyros). This “positive” BAL displayed neutralizing activity, completely blocking luciferase induction in response to stimulation with IFN-α2, but not IFN-β (Figure S1D). By purifying the IgG, we were able to show that the neutralizing activity was IgG-mediated (Figure S1E).

### Auto-Abs Neutralizing IFN-α2 and/or IFN-ω in the BAL of at Least 10% of Patients with Life-Threatening COVID-19 Pneumonia

We then assessed the neutralizing capacity of these anti-IFN-α and anti-IFN-ω IgG auto-Abs present in the BAL of patients with life-threatening COVID-19 pneumonia. The median time from the onset of COVID-19 symptoms to BAL sampling was 15 days (interquartile range, IQR: 9–21 days), and that from ICU admission to BAL sampling was 7 days (IQR: 4 to 12 days) (Table 1). Given the ~ 100-fold dilution of the ELF in the BAL ([47] and Table S1), we tested the neutralizing capacity of these antibodies exclusively with low concentrations (100 pg/mL) of IFN-α2 or IFN-ω (corresponding to a neutralizing capacity of ~ 10 ng/mL by the ELF). We tested 415 individuals and found that 45 (11%) and 37 (9%) had auto-Abs neutralizing IFN-α2 and IFN-ω, respectively, in their BAL (Fig. 1D and Table 3); 54 (13%) had auto-Abs neutralizing IFN-α2 and/or IFN-ω, and 28 (7%) had auto-Abs neutralizing both IFN-α2 and IFN-ω. As reported for plasma auto-Abs, the auto-Abs neutralizing IFN-α2 in the BAL were also able to neutralize the other 12 type I IFN subtypes (Figure S1F) [8]. We also tested the BAL for the presence of auto-Abs neutralizing IFN-β (10 ng/mL, as no luciferase induction was observed with lower concentrations). We identified five (1%) patients with auto-Abs neutralizing IFN-β: three (0.7%) had auto-Abs neutralizing IFN-α2, IFN-ω, and IFN-β, and two (0.5%) had auto-Abs neutralizing IFN-α2 and IFN-β (Fig. 1D and Table 3). Finally, we assessed the correlation between the presence of auto-Abs neutralizing type I IFNs in plasma and in BAL. In total, 95 (mean age: 65 years, 72% men) patients had paired plasma and BAL samples. Relative to the other patients of the cohort, these 95 patients were older, but the proportion of male patients, rates of death and auto-Abs against IFN-α2 and/or IFN-ω, and the duration of invasive ventilation and of the stay in ICU were similar (Table S2). Seven of these patients (7%) had auto-Abs neutralizing IFN-α2 in both the plasma and the BAL, and one (1%) had auto-Abs neutralizing IFN-α2 in the plasma but not in the BAL. Four (4%) individuals had auto-Abs neutralizing IFN-ω in both BAL and plasma. Two (2%) individuals had auto-Abs neutralizing IFN-ω in plasma but not BAL. Two (2%) individuals had auto-Abs neutralizing IFN-β only in the plasma (Fig. 1E). Thus, auto-Abs neutralizing type I IFNs are present in the alveolar space of at least 10% of patients with life-threatening COVID-19 pneumonia.

**Table 3:** Number and proportion of patients with auto-Abs neutralizing type I IFNs in bronchoalveolar lavage

Type and concentration of type I IFNs neutralized by BAL diluted 1:5	Number (%) of patients with neutralizing activity
Anti–IFN-α2 and/or anti–IFN-ω auto-Abs (100 pg/mL)	54 (13%)
Anti–IFN-α2 and anti–IFN-ω auto-Abs (100 pg/mL)	28 (7%)
Anti–IFN-α2 auto-Abs (100 pg/mL)	45 (11%)
Anti–IFN-ω auto-Abs (100 pg/mL)	37 (9%)
Anti–IFN-β auto-Abs (10 ng/mL)	5 (1%)
Anti–IFN-α2 (100 pg/mL), anti–IFN-ω (100 pg/mL) and anti–IFN-β auto-Abs (10 ng/mL)	3 (1%)

### Similar Outcome and Alveolar Inflammation in Patients with Life-Threatening COVID-19 Pneumonia with and Without Auto-Abs Against Type I IFNs in the BAL

Mortality was similar in patients with life-threatening COVID-19 pneumonia with and without auto-Abs against type I IFNs (Table 1). The presence of auto-Abs against type I IFNs was not associated either with the duration of invasive ventilation or length of ICU stay (Table 1). It has been reported that impaired type I IFN immunity in the first few days of SARS-CoV-2 infection, due to auto-Abs against type I IFNs or inborn errors of type I IFN immunity, results in excessive inflammation from the second week of infection onward [1]. We therefore assessed the impact of auto-Abs neutralizing IFN-α2 and/or IFN-ω on the expression of inflammatory biomarkers in the BAL. We assessed the concentration of 59 biomarkers (Table S3) in the BAL of 11 (mean age: 68 years, 100% men) patients with auto-Abs neutralizing IFN-α2 and/or IFN-ω in BAL and 117 (mean age: 64 years, 71% men) patients without such auto-Abs. All these patients had life-threatening COVID-19 pneumonia. Principal component analysis (PCA) revealed no difference in clustering between patients with and without auto-Abs against type I IFNs (Fig. 1F). Moreover, only three biomarkers (MIP1α, MIP1β, and TRAIL) were present at significantly higher concentrations (with a log_2_FC of at least 1 and a *p*-value < 0.05) in the BAL of patients with auto-Abs against type I IFNs than in the BAL of patients without such antibodies (Fig. 1G). Overall, these results suggest that, in patients with life-threatening COVID-19 pneumonia, inflammation in the alveolar space is similar, at least for the biomarkers evaluated, between those with and without auto-Abs against type I IFNs.

## Discussion

We report that at least 10% of the patients with life-threatening COVID-19 pneumonia tested have auto-Abs neutralizing high concentrations (10 ng/mL) of type I IFNs in the lower respiratory tract during SARS-CoV-2 infection. The neutralizing activity was mediated by the IgG fraction of the BAL, suggesting that it was not IgA-mediated. All patients with auto-Abs neutralizing type I IFNs in their BAL for whom paired plasma samples were available also had these auto-Abs in their plasma, whereas a few (2%) patients had auto-Abs detected only in plasma. These observations suggest that the IgG auto-Abs against type I IFNs circulating in the plasma can reach the alveolar space. The ELF was estimated to be diluted ~ 100-fold in the BAL samples tested. We may not, therefore, have been able to detect auto-Abs neutralizing lower concentrations of type I IFNs. The prevalence of auto-Abs neutralizing type I IFNs in the lower respiratory tract during SARS-CoV-2 infection may therefore be greater than 10%, perhaps closer to the 15% documented for blood [7, 8]. Like auto-Abs neutralizing type I IFNs in the nasopharyngeal mucosa [42], auto-Abs in the BAL probably contribute to the spread of the virus to and within the lower respiratory tract. In the nasopharyngeal mucosa, these antibodies are associated with a decrease in type I/III IFN-dependent ISG induction [42]. They may also impair antiviral type I IFN immunity in the alveolar space, leading to life-threatening COVID-19 pneumonia. We know that these auto-Abs are present in the plasma before SARS-CoV-2 infection [7, 15]. Moreover, immunoglobulins, including IgG, are present in the epithelial lining fluid of healthy individuals [47]. Auto-Abs neutralizing type I IFNs are, thus, probably present in the alveolar space before SARS-CoV-2 infection, although it is not possible to draw definitive conclusions on this point because we had no access to BAL samples obtained from these patients before infection. We also cannot exclude the possibility that these antibodies cross the mucosae as a consequence of viral spread, although our findings suggest that they can reach the alveolar space without the need for alveolar hemorrhage. Regardless of the timing and mechanism of their arrival in the alveolar space, these auto-Abs probably impair local type I IFN immunity, thereby contributing to hypoxemic COVID-19 pneumonia. In line with our previous single-cell RNA-sequencing (scRNAseq) analysis on blood from patients with life-threatening COVID-19 pneumonia [23], the similar alveolar inflammation profiles observed in patients with life-threatening COVID-19 with and without auto-Abs against type I IFNs further suggest that impaired type I IFN immunity is a general pathogenic mechanism.

## Supplementary Information

Below is the link to the electronic supplementary material.

Supplementary file1 (A) Bar plot of the distribution of age, sex, and death for the patients with life-threatening COVID-19 studied (*N* = 415). (B) Hemoglobin concentration (mg/dL) in the BAL of patients with life-threatening COVID-19, with or without cytologically confirmed alveolar hemorrhage (AH) (*N* = 3 and 1, respectively), and with or without auto-Abs neutralizing IFN-α2 and/or IFN-ω (AAB—*N* = 4 and 2, respectively). (C) Relative luciferase activity (RLA) without stimulation, or after stimulation with IFN-α2 (100 pg/mL or 10 ng/mL) or IFN-β (10 ng/mL), with or without monoclonal (mAb) anti-human IFN-α2 IgG (1 μg/mL or 10 μg/mL) in the presence of 1:5 PBS or a “negative” bronchoalveolar lavage (BAL) (no anti-IFN-α2 and no anti-IFN-ω IgG detected in the BAL or corresponding plasma). (D) RLA after stimulation with IFN-α2 (100 pg/mL) or IFN-β (10 ng/mL), in the presence of a 1:5 dilution of BAL with anti-IFN-α2 but no anti- IFN-β IgG (BAL with AAB) or without anti-IFN-α2 or anti-IFN-β IgG (BAL without AAB), normalized against the RLA obtained without stimulation in the presence of a 1:5 dilution of BAL. (E) IgG purification experiment with BAL samples from six patients with life-threatening COVID-19, four of which were capable of neutralizing 100 pg/mL IFN-α2 but not 10 ng/mL IFN-β (BAL with AAB), the other two BAL samples being unable to neutralize 100 pg/mL IFN-α2 as well as 10 ng/mL IFN-β (BAL without AAB). The RLA ratio is shown after stimulation with 100 pg/mL IFN-α2 or 10 ng/mL IFN-β, in presence of the whole BAL, the IgG-depleted fraction of the BAL, or the IgG-positive eluted fraction of the BAL (IgG^+^). (F) RLA after stimulation with all individual subtypes of IFN-α at a concentration of 1 ng/mL, with a 1:10 dilution of plasma from three healthy controls (negative controls – E1, E2, and E3) and a patient with APS-1 (positive control – Ctrl. +), or with a 1:5 dilution of BAL from five patients with life-threatening COVID-19 (B1 to B5) capable or incapable of neutralizing IFN-α2 and IFN-ω (PDF 228 KB)

Supplementary file2 (DOCX 18 kb)

## Data Availability

All raw and processed data are available upon request from the corresponding authors under a material/data transfer agreement.
