# Supplementary figures and images for: Autoantibodies Neutralizing Type I IFNs in the Bronchoalveolar Lavage of at Least 10% of Patients During Life-Threatening COVID-19 Pneumonia
Source: J Clin Immunol. 2023 May 20;43(6):1093–103. doi: 10.1007/s10875-023-01512-9 (PMC10199445; doi:10.1007/s10875-023-01512-9)

Figure S1

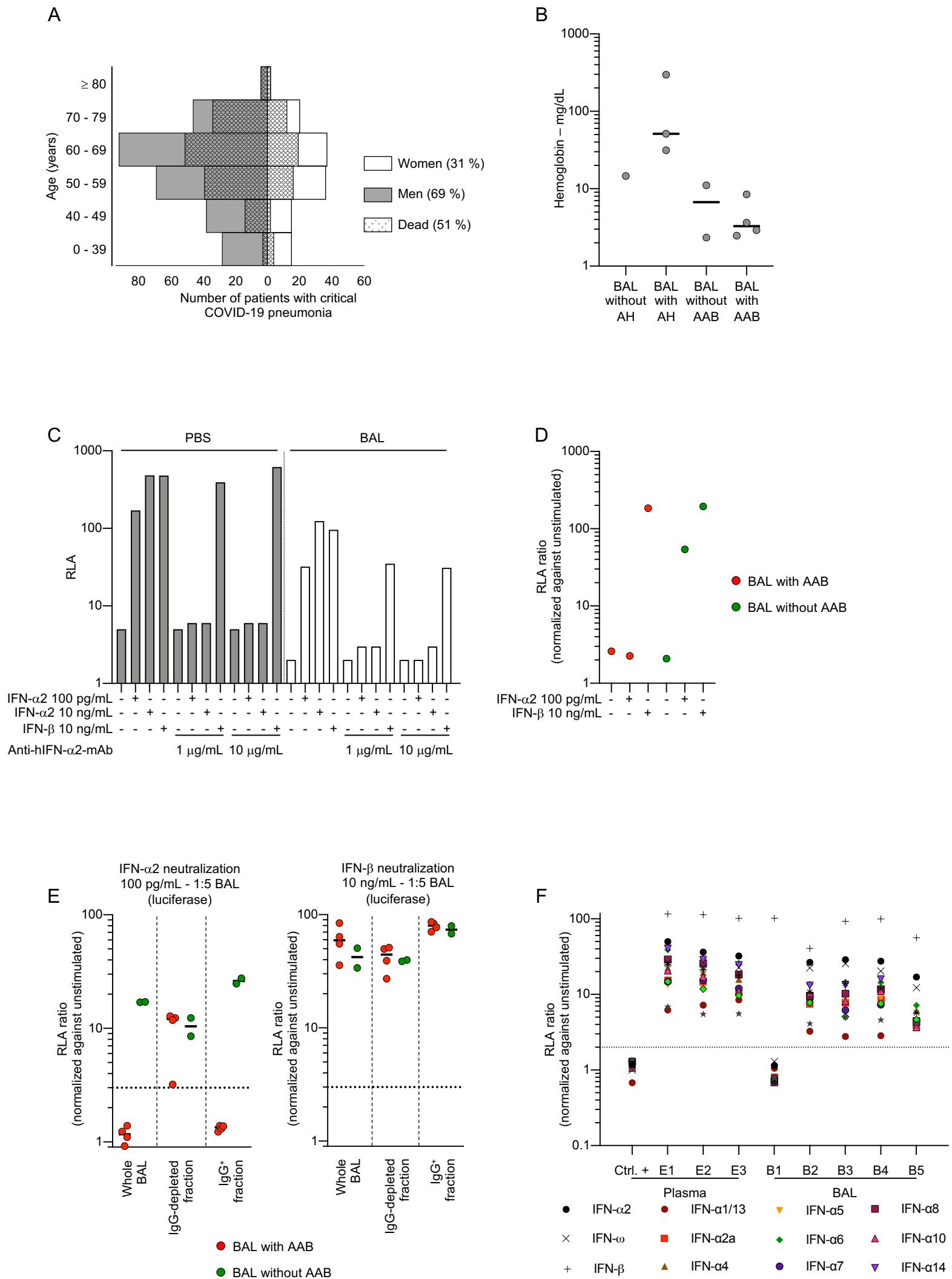

Supplement: Supplementary file 1 — Supplementary file1 (A) Bar plot of the distribution of age, sex, and death for the patients with life-threatening COVID-19 studied (N = 415). (B) Hemoglobin concentration (mg/dL) in the BAL of patients with life-threatening COVID-19, with or without cytologically confirmed alveolar hemorrhage (AH) (N = 3 and 1, respectively), and with or without auto-Abs neutralizing IFN-α2 and/or IFN-ω (AAB—N = 4 and 2, respectively). (C) Relative luciferase activity (RLA) without stimulation, or after stimulation with IFN-α2 (100 pg/mL or 10 ng/mL) or IFN-β (10 ng/mL), with or without monoclonal (mAb) anti-human IFN-α2 IgG (1 μg/mL or 10 μg/mL) in the presence of 1:5 PBS or a “negative” bronchoalveolar lavage (BAL) (no anti-IFN-α2 and no anti-IFN-ω IgG detected in the BAL or corresponding plasma). (D) RLA after stimulation with IFN-α2 (100 pg/mL) or IFN-β (10 ng/mL), in the presence of a 1:5 dilution of BAL with anti-IFN-α2 but no anti- IFN-β IgG (BAL with AAB) or without anti-IFN-α2 or anti-IFN-β IgG (BAL without AAB), normalized against the RLA obtained without stimulation in the presence of a 1:5 dilution of BAL. (E) IgG purification experiment with BAL samples from six patients with life-threatening COVID-19, four of which were capable of neutralizing 100 pg/mL IFN-α2 but not 10 ng/mL IFN-β (BAL with AAB), the other two BAL samples being unable to neutralize 100 pg/mL IFN-α2 as well as 10 ng/mL IFN-β (BAL without AAB). The RLA ratio is shown after stimulation with 100 pg/mL IFN-α2 or 10 ng/mL IFN-β, in presence of the whole BAL, the IgG-depleted fraction of the BAL, or the IgG-positive eluted fraction of the BAL (IgG+). (F) RLA after stimulation with all individual subtypes of IFN-α at a concentration of 1 ng/mL, with a 1:10 dilution of plasma from three healthy controls (negative controls – E1, E2, and E3) and a patient with APS-1 (positive control – Ctrl. +), or with a 1:5 dilution of BAL from five patients with life-threatening COVID-19 (B1 to B5) capable or incapable of n [file 10875_2023_1512_MOESM1_ESM.pdf]
