# Supplementary material for: Autoantibodies Neutralizing Type I IFNs in the Bronchoalveolar Lavage of at Least 10% of Patients During Life-Threatening COVID-19 Pneumonia
Source: J Clin Immunol. 2023 May 20;43(6):1093–103. doi: 10.1007/s10875-023-01512-9 (PMC10199445; doi:10.1007/s10875-023-01512-9)
Supplement: Supplementary file 2 — Supplementary file2 (DOCX 18 kb) [file 10875_2023_1512_MOESM2_ESM.docx]

**Supplemental material**

**Table S1. Urea concentration in the bronchoalveolar lavage and plasma, and estimated dilution of the epithelial lining fluid**

|  | **BAL** | **Plasma** | **ELF dilution** |
| --- | --- | --- | --- |
| **Sp. ID** | **μmol/L** | **mmol/L** |  |
| **A** | 13.9 | 4.2 | 302 |
| **B** | 28.4 | 6.1 | 215 |
| **C** | 22.0 | 4.0 | 182 |
| **D** | 39.4 | 8.9 | 226 |
| **E** | 64.6 | 6.1 | 94 |

Epithelial lining fluid (ELF) dilution was estimated as follows: ELF dilution= [urea in the BAL]/[urea in the plasma], assuming that [urea in the ELF]=[urea in the plasma], [47]

**Table S2. Comparison of the clinical characteristics of patients with and without paired plasma and BAL samples**

|  | **Patients without paired plasma and BAL samples** | **Patients with paired plasma and BAL samples** | ***P*-value*** |
| --- | --- | --- | --- |
| Age (years) | 57 (48 – 65) | 66 (60 - 72) | <0.0001 |
| Male | 213 (68%) | 64 (72%) | 0.49 |
| Death | 154 (50%) | 48 (54%) | 0.55 |
| Anti–IFN-α2 and/or anti–IFN-ω auto-Abs (100 pg/mL) | 44 (14%) | 10 (11%) | 0.54 |
| Median duration of ICU stay (days) | 31 (18 – 50) | 24 (14 – 44) | 0.35 |
| Median duration of invasive ventilation (days) | 27 (16 – 44) | 20 (11 – 37) | 0.16 |
| Time from ICU admission to BAL (days) | 8 (5 – 13) | 7 (4 – 11) | 0.79 |

Data are reported as *n, n* (%), or median (interquartile range); * The two groups were compared in *t*- or chi-squared tests, with a *P*-value < 0.05 considered significant.

**Table S3. List of the 59 biomarkers assessed in the BAL of 11 patients with auto-Abs neutralizing IFN-α2 and/or IFN-ω in BAL and 117 patients without such auto-Abs**

| Angiopoietin-1 | IL-5 |
| --- | --- |
| Angiopoietin-2 | IL-6 |
| CD40 ligand | IL-7 |
| E selectin | IP-10 |
| EGF | MCP-1 |
| ENPP 2 | MIP1α |
| Eotaxin | MIP1β |
| FGF | MIP3α |
| Flt 3 ligand | MIP-3β |
| Fractalkine | MMP-1 |
| G CSF | MMP-8 |
| GM CSF | Osteopontin |
| Granzyme B | P-selectin |
| GRO-α | PD-L1 |
| GRO-β | PDGF-AA |
| IFN-α | PDGF-AB-BB |
| IFN-β | Periostin |
| IFN-γ | RAGE |
| IL-10 | Rantes |
| IL-12p70 | SP-D |
| IL-13 | TGF-α |
| IL-15 | Thrombomodulin |
| IL-17A | TNF RI |
| IL-17E | TNF |
| IL-1α | TRAIL |
| IL-1β | Urokinase |
| IL-2 | VCAM-1 |
| IL-3 | VEGF |
| IL-33 | vWF |
| IL-4 |  |
